# A 5'-proximal Stem-loop Structure of 5' Untranslated Region of Porcine Reproductive and Respiratory Syndrome Virus Genome Is Key for Virus Replication

**DOI:** 10.1186/1743-422X-8-172

**Published:** 2011-04-15

**Authors:** Jiaqi Lu, Fei Gao, Zuzhang Wei, Ping Liu, Changlong Liu, Haihong Zheng, Yanhua Li, Tao Lin, Shishan Yuan

**Affiliations:** 1 Department of Swine Infectious Diseases, Shanghai Veterinary Research Institute, Chinese Academy of Agricultural Sciences, Shanghai 200241, China; 2 College of Veterinary Medicine, Nanjing Agricultural University, Nanjing 210095, China

## Abstract

**Background:**

It has been well documented that the 5' untranslated region (5' UTR) of many positive-stranded RNA viruses contain key *cis*-acting regulatory sequences, as well as high-order structural elements. Little is known for such regulatory elements controlling porcine arterivirus replication. We investigated the roles of a conserved stem-loop 2 (SL2) that resides in the 5'UTR of the genome of a type II porcine reproductive and respiratory syndrome virus (PRRSV).

**Results:**

We provided genetic evidences demonstrating that 1) the SL2 in type II PRRSV 5' UTR, N-SL2, could be structurally and functionally substituted by its counterpart in type I PRRSV, E-SL2; 2) the functionality of N-SL2 was dependent upon the G-C rich stem structure, while the ternary-loop size was irrelevant to RNA synthesis; 3) serial deletions showed that the stem integrity of N-SL2 was crucial for subgenomic mRNA synthesis; and 4) when extensive base-pairs in the stem region was deleted, an alternative N-SL2-like structure with different sequence was utilized for virus replication.

**Conclusion:**

Taken together, we concluded that the phylogenetically conserved SL2 in the 5' UTR was crucial for PRRSV virus replication, subgenomic mRNA synthesis in particular.

## Background

Porcine reproductive and respiratory syndrome virus (PRRSV) is a member of the family *Arteriviridae *that belongs to the order *Nidovirales*, which also includes *Coronaviridae *and *Roniviridae*. Arterivirus contains a positive-sense RNA genome that is 5' capped and 3' polyadenylated [1]. A set of 3' co-terminal, nested subgenomic (sg) mRNAs are synthesized for expression of eight structural proteins, including the recently described ORF5a [2,3]. The sg mRNA shares the common genomic 5' leader sequence, the whole 5' untranslated region (UTR) for PRRSV, which links with various 3' proximal genomic regions that are referred to as "mRNA bodies" [4]. The crucial control elements for the fusion of leader and bodies are the transcription-regulating sequences (TRSs) that are present at the 3' end of the leader sequence (leader TRS) and the 5' end of the coding sequence for each ORF (body TRSs) [5]. This discontinuous RNA transcription most likely happens during synthesis of the negative-strand sg mRNA template, for which the base paring between the leader TRS (in the plus strand) and the complement of the body TRSs (in the nascent minus strand) is critical [6-9]. PRRS is the biggest threat to the swine industry worldwide, especially in developing countries, where loss from PRRS outbreaks has been huge in spite of massive use of PRRS vaccine [10,11]. One of the major challenges for PRRS control is the lack of knowledge about the biology of PRRSV, in particular, the details of virus replication.

It is well established that the 5' UTRs of many positive-stranded RNA viruses contain key *cis*-acting regulatory sequences, as well as high-order structural elements. For coronavirus, at least three major stem-loops, SL1, SL2 and SL4, conserved in nine coronaviruses, have been proved to play crucial roles in virus replication [12]. Furthermore, it has been demonstrated that the SL2 is required for mouse hepatitis virus (MHV) replication and sg mRNA synthesis. The SL2 typically contains a pentaloop (C_47_-U_48_-U_49_-G_50_-U_51 _in MHV) stacked on a 5-bp stem. This "U-turn like" conformation is important but rather plastic [12,13]. Moreover, the 5' UTRs between different groups of coronaviruses have an arrays of conserved stem-loops, some of which are inter-changeable among different viruses, such structural elements may facilitate the presentation of the consensus leader TRS sequence accessible for discontinuous RNA transcription [14,15]. For *Picoronaviridae *members, e.g. Aichi virus, three stem-loops at the 5' end of the genome are crucial for viral RNA replication [16,17]. In addition, a pseudoknot structure formed by RNA-RNA tertiary interaction between two stem-loops in the 5'-terminal genomic region is crucial for negative-strand RNA synthesis for Aichi viruses [16]. Moreover, the 5' leader sequences of poliovirus can form a cloverleaf structure and play a direct role in regulating the viral positive and negative-strand RNA replication [18]. It has also been reported that the 5' end of the genome of Sindbis virus contains *cis*-acting elements that regulate positive and negative-strand RNA synthesis [19]. Little is known for regulatory elements controlling porcine arterivirus replication.

It is believed that the PRRSV 5' UTR is also crucial for viral RNA synthesis, yet many details of the mechanism that regulate the genome replication and discontinuous subgenomic transcription remain to be elucidated. There are two different genotypes of PRRSV, type I (European-type, EU) [20] and type II (American-type, NA) [21], which exhibit approximately 60% nucleotide sequence identity [22,23]. PRRSV 5' UTR differs in sequence length, 220 and 190 nt for EU and NA types, respectively, and sharing only 50% genetic identity [24,25]. Choi et al. (2006) have reported that the first seven nucleotides of the PRRSV 5' UTR are nonessential for virus viability. However, the 5' UTR deletion mutant contains a variety of compensatory foreign 5' AU-rich sequences [26]. Although the primary sequences between the two genotypes of PRRSV are distantly related, the predicted secondary structures display significant similarity [4]. Six stem-loop structures were predicted for the 5'-proximal region for type I (nt 1-280) and type II (1-246) PRRSV, which are relevant to the inter-genotypically conserved 5' UTR domains. One of the stem-loops is the leader TRS-containing hairpin (LTH), first identified in the 5' proximal region of the prototypic equine arteritis virus (EAV), is apparently conserved among other members of the arteriviruses and even in some coronaviruses [4]. The group of Snijder has demonstrated that the LTH and its immediate flanking sequences are crucial for sg mRNAs synthesis, with little effect on genome replication and translation [4,27]. There is no information about the structure and function relationship of the highly structured 5' UTR of other three known arteriviruses, which share little genome-wide nucleotide sequence identity.

To identify the *cis*-acting sequences and structural elements controlling porcine arterivirus replication, we conducted reverse genetic manipulation to investigate the roles of a conserved stem-loop 2 (SL2) that resides in 5' UTR of the PRRSV genome. *In silico *analysis of two types of PRRSV 5' UTRs suggested that SL2 was an inter-genotypically conserved stem-loop structure. The SL2 of type II PRRSV (N-SL2) could be structurally and functionally replaced by the counterpart of type I PRRSV, designated as E-SL2. Site-specific mutagenesis revealed that the loop size of N-SL2 was irrelevant to the synthesis of PRRSV sg mRNAs, for which the N-SL2 stem structure was crucial. Serial base-pair deletions in the stem region confirmed that N-SL2 could be possibly linked with the viral sg mRNA transcription level. Taken together, we provided genetic evidence demonstrating that SL2 is a key regulatory structural element for PRRSV replication, particularly sg mRNA synthesis.

## Methods

### Cells and viruses

Baby hamster kidney (BHK-21, ATCC CCL10) cells were grown and maintained in Eagle's Minimum Essential Medium (EMEM; Invitrogen) supplemented with 10% fetal bovine serum (FBS; Invitrogen). MARC-145 cells (ATCC, Manassas, VA, USA) were grown at 37°C and 5% CO_2 _in EMEM supplemented with 10% FBS, and maintained EMEM with 2% FBS. All viruses rescued from the type II PRRSV infectious clone pAPRRS and derivatives were propagated in MARC-145 cells as described previously [28].

### Virus strains and viral sequences

The nucleotide sequences of PRRSV 5' UTRs were aligned by use of the DNASTAR v7.1 program (Lasergene Package). The genomic sequences were retrieved from GenBank. Five available strains of type I PRRSV were included: LV (GenBank: M96262), HKEU16 (EU076704), LV421 (AY588319), SD0108 (DQ489311), and EuroPRRSV (AY366525). Overwhelming number of type II PRRSV genomic sequences have been deposited into GenBank during the last five years, most of which are highly-pathogenic Chinese strains. To decrease the sequence bias of the PRRSV isolates, nine type II PRRSV sequences were used to generate the consensus 5' proximal sequences corresponding to the first 246 nucleotides of APRRSV (GQ330474), the backbone virus used throughout this study. Also included virus strains are the prototypic VR2332 (AY150564), SP (AF184212), BJ-4 (AF331831), CH1A (AY032626), HB-2 (AY262352), P129 (AF494042), JX143 (EF488048), and JXA1 (EF112445). For RNA structure analysis, other arteriviruses were also included as SHFV strain LVR 42-0/M6941 (NC_003092), and LDV strain Plagemann (NC_001639). The coronaviruses including poecine epidemic diarrhea virus (PEDV) strain CV777 (NC_003436), MHV strain A59 (NC_001846) and Bovine coronavirus (BCoV) strain Quebec (AF220295), were also adopted for similarity analysis.

### PCR-based site-directed mutagenesis

**Figure 1:**
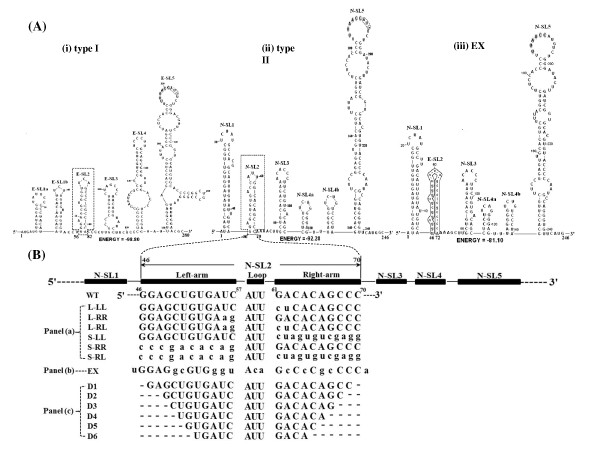
**An inter-genotypically conserved RNA secondary structure models of the 5'-proximal genomic region of PRRSV, based on the consensus sequences generated by sequence lineup (Lasergene Package)**. (A) Predicted RNA structure by MFold for different genotypes and chimeric sequences. (i) RNA secondary structure prediction of the consensus 5'-proximal 280 nt of type I PRRSV genome from five available type I PRRSV sequences. (ii) Predicted RNA secondary structure of the consensus 5'-proximal 246 nt generated by comparison of nine type II PRRSV genomes. Stem-loop 2 (SL2) in both models are highlighted by the dashed box. The leader TRS and start codon for ORF1a in two models are shown by gray shading and solid boxes separately. Stem-loop structures are designated as E-SL1-5 for type I PRRSV and N-SL1-5 for type II, respectively. (iii) Predicted secondary structure of the 5'-proximal 246 nt of mutant EX generated by substituting N-SL2 with E-SL2. The mutant region in EX is highlighted by solid box and lowercase. (B) Schematic drawing sequence location of type II PRRSV stem-loops, represented by black boxes. Parental (WT) N-SL2 sequence from nt 46-70 (GQ330474) was shown, based on which mutations (lowercase) were made. Dashed lines represent stem base-pair deletions in the mutant plasmids D1-D6.

For the convenience of genetic manipulations, a shuttle plasmid pCBSA was generated by truncating the genomic sequences in the full-length cDNA clone pAPRRS by *Sph *I, in between the CMV transcription start site and the 5' UTR and *Afl *II (nt 1688 in APRRSV) and cloned into pBluscript SK+ vector. The desired mutations were introduced into pCBSA by the Quik-Change site-directed PCR mutagenesis method (Stratagene), and the primers used for PCR mutagenesis are listed in Table 1. The fragments carrying the verified mutations were then transferred into the corresponding region of the pAPRRS. Specifically, the 3-nt loop of N-SL2 was enlarged to 8 nt by substituting G_61_A_62 _(GQ330474) with C_61_U_62 _(Figure 1B panel a), and the full-length clone was designated as mutant L-LL. In the similar manner, mutant L-RR was generated by replacing two nucleotides U_56_C_57 _with A_56_G_57_. Subsequently, L-RL was generated by combining the mutations in both L-LL and L-RR, so that the 3-nt loop and the one nucleotide (A_55_) budge were restored.

**Table 1:** Primers for this study

Name	Sequence	Application
**F-L-LL**	5'-GTATTGTCAggagctgtgaAGattgacacagcccAAAGCTTGCTGCACAG-3'	PCR mutagenesis for mutant L-LL
**R-L-LL**	5'-CTGTGCAGCAAGCTTTgggctgtgtcaatCTtcacagctccTGACAATAC-3'	
**F-L-RR**	5'-GTATTGTCAggagctgtgatcattCTcacagcccAAAGCTTGCTGCACAG-3'	PCR mutagenesis for mutant L-RR
**R-L-RR**	5'-CTGTGCAGCAAGCTTTgggctgtgAGaatgatcacagctccTGACAATAC-3'	
**F-L-RL**	5'-GTATTGTCAggagctgtgaAGattCTcacagcccAAAGCTTGCTGCACAG-3'	PCR mutagenesis for mutant L-RL
**R-L-RL**	5'-CTGTGCAGCAAGCTTTgggctgtgAGaatCTtcacagctccTGACAATAC-3'	
**F-S-LL**	5'-GCCTTGACATTTGTATTGTCAggagctgtgatcattctgtgtcgggAAAGCTTGCTGCACAGAAAC-3'	PCR mutagenesis for mutant S-LL
**R-S-LL**	5'-GTTTCTGTGCAGCAAGCTTTcccgacacagaatgatcacagctccTGACAATACAAATGTCAAGGC-3'	
**F-S-RR**	5'-GCCTTGACATTTGTATTGTCAccacgacacaagattgacacagcccAAAGCTTGCTGCACAGAAAC-3'	PCR mutagenesis for mutant S-RR
**R-S-RR**	5'-GTTTCTGTGCAGCAAGCTTTgggctgtgtcaatcttgtgtcgtggTGACAATACAAATGTCAAGGC-3'	
**F-S-RL**	5'-GCCTTGACATTTGTATTGTCAcccgacacagattctagtgtcgaggAAAGCTTGCTGCACAGAAAC-3'	PCR mutagenesis for mutant S-RL
**F-S-RL**	5'-GTTTCTGTGCAGCAAGCTTTcctcgacactagaatctgtgtcgggTGACAATACAAATGTCAAGGC-3'	
**F-EX**	5'-GCCTTGACATTTGTATTGTCAtggaggcgtgggtacagccccgccccaAAAGCTTGCTGCACAGAAAC-3'	PCR mutagenesis for mutant EX
**R-EX**	5'-GTTTCTGTGCAGCAAGCTTTtggggcggggctgtacccacgcctccaTGACAATACAAATGTCAAGGC-3'	
**F-D1**	5'-GACATTTGTATTGTCAgagctgtgatcattgacacagccAAAGCTTGCTGCACAG-3'	PCR mutagenesis for mutant D1
**R-D1**	5'-CTGTGCAGCAAGCTTTggctgtgtcaatgatcacagctcTGACAATACAAATGTC-3'	
**F-D2**	5'-GACATTTGTATTGTCAgctgtgatcattgacacagcAAAGCTTGCTGCACAG-3'	PCR mutagenesis for mutant D2
**R-D2**	5'-CTGTGCAGCAAGCTTTgctgtgtcaatgatcacagcTGACAATACAAATGTC-3'	
**F-D3**	5'-GCCTTGACATTTGTATTGTCActgtgatcattgacacagAAAGCTTGCTGCACAGAAAC-3'	PCR mutagenesis for mutant D3
**R-D3**	5'-GTTTCTGTGCAGCAAGCTTTctgtgtcaatgatcacagTGACAATACAAATGTCAAGGC-3'	
**F-D4**	5'-GCCTTGACATTTGTATTGTCAtgtgatcattgacacaAAAGCTTGCTGCACAGAAAC-3'	PCR mutagenesis for mutant D4
**R-D4**	5'-GTTTCTGTGCAGCAAGCTTTtgtgtcaatgatcacaTGACAATACAAATGTCAAGGC-3'	
**F-D5**	5'-GCCTTGACATTTGTATTGTCAgtgatcattgacacAAAGCTTGCTGCACAGAAAC-3'	PCR mutagenesis for mutant D5
**R-D5**	5'-GTTTCTGTGCAGCAAGCTTTgtgtcaatgatcacTGACAATACAAATGTCAAGGC-3'	
**F-D6**	5'-GCCTTGACATTTGTATTGTCAtgatcattgacaAAAGCTTGCTGCACAGAAAC-3'	PCR mutagenesis for mutant D6
**R-D6**	5'-GTTTCTGTGCAGCAAGCTTTtgtcaatgatcaTGACAATACAAATGTCAAGGC-3'	
**Qst**	5'-GAGTGACGAGGACTCGAGCGCATGCTTTTTTTTTTTTTT-3'	RT for cDNA preparation
**F-6**	5'-GTATAGGTGTTGGCTCTATGC-3'	(-) gRNAs analysis
**R-683**	5'-GGAGCGGCAGGTTGGTTAACACG-3'	(-) gRNAs analysis
**F-12**	5'-GTGTTGGCTCTATGCCTTGAC-3'	(-) gRNAs analysis, sg mRNA7 analysis
**R-343**	5'-ATAGAATAGGCCCAGCACCCC-3'	(-) gRNAs analysis
**R-15284**	5'-CTCCACAGTGTAACTTATCCTCC-3'	sg mRNA7 analysis
**F-actin**	5'-CCCATCTATGAGGGCTACGC-3'	β-actin analysis
**R-actin**	5'-TTTGATGTCACGCACAATTTC-3'	β-actin analysis

Prefixes: F, forward primer; R, reverse primer; RT, reverse transcription; (-) gRNAs, negativ-strand genomic RNA; sg, subgenomic. The lowercase represent different sequences compared the APRRSV (GQ330474) in N-SL2.

To investigate the functionality of the stem, the N-SL2 stem was disrupted by replacing the right arm sequence with the reverse sequences of the left arm (3' -G_70_GAGCUGUGAUC_61_-5', Figure 1B panel a), designated as mutant S-LL. In the same manner, the left stem was replaced with the reverse sequence of the right arm, thereby generating mutant S-RR. Subsequently, the mutated arms were combined together in S-RL, in which the stem was predicted to be restored by MFold prediction [29].

To investigated if the equivalent E-SL2 can replace N-SL2, the consensus E-SL2 sequence of the type I PRRSV 5' UTRs (_56_uGGAGgcGUGgguAcaGcCcCgcCCCa_82_, heterologous sequences compared to APRRSV are shown in lowercase) was used for substitution of N-SL2, and named as EX (Figure 1B panel b). To investigate the significance of the length and/or stability of the N-SL2 stem, a panel of serial base-pair deletions of the stem from the bottom of the N-SL2 stem were created, thereby generating mutants D1-D6 (Figure 1B panel c), respectively.

**Figure 2:**
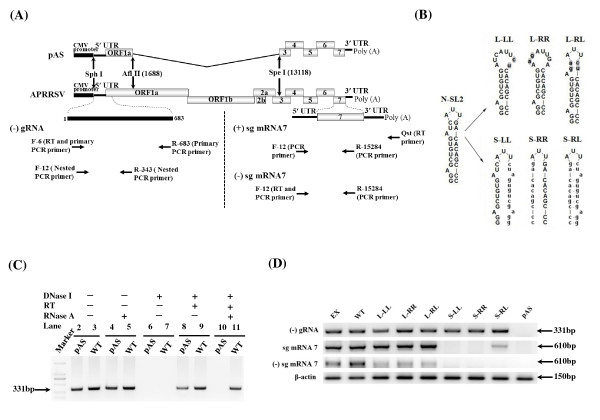
**Mutational analysis of the predicted stem-loop structure in the N-SL2**. (A) Strategic representation of RT-PCR used to detect (-) gRNA, (+) sg mRNA7 and (-) sg mRNA7. The positions are according to APRRSV stain (GenBank: GQ330474) and all primer sequences are listed in Table 1. pAS was a non-replicative control which was absence of gene ORF1a and ORF1b (1688-13118) in full-length cDNA clone. (B) Schematic representation of the mutations introduced into the N-SL2 structure. The loop was enlarged as described in Figure 1, and mutants L-LL and L-RR were generated by overlapping PCR mutagenesis. L-RL was generated by combining the right and left arm sequences of the L-LL and L-RR, respectively, such that the overall structure of N-SL2 was restored. All the mutated nucleotides (lowercase) are highlighted in gray shading. The stem mutants, S-LL and S-RR, were generated by overlapping PCR such that one arm sequence was replaced with that of the opposite arm. The double mutant, S-RL, was generated by combining the mutations in the left and right arms such that the overall structure was restored. All mutant sequences are shown as lowercase. (C) RT-PCR of RNAs extracted from pAS and WT transfected cells at 24 hours after transfection. DNase I and RNase A were used to omit template DNA and the reverse transcriptase. The primers were nested RT-PCR primers as same as (-) gRNA detection. A 2-kbp ladder was used as a molecular size marker. The numbers indicated the lane No. (D) RT-PCR analysis of the mutants. Total cellular RNAs were extracted from mutant plasmids-transfected from BHK-21 cells at 24 hours post-transfection. β-actin is a marker for the level of intracellular RNA isolation, and pAS is a non-replicative control.

As a non-replicative plasmid control, pAS was also constructed by deleting nt 1688-13118 of the pAPRRS via double digestion with restriction enzyme *Afl *II and *Spe *I, followed by filling-in by Klenow DNA polymerase and self-ligation (Figure 2A). Because that the majority of the non-structural protein and the minor envelope proteins coding regions were deleted, the CMV promoter-driven pAS is not replicative in the DNA transfected cells. All of the plasmids were verified using restricted enzymatic mapping and nucleotide sequencing (Shanghai Sangon Inc.).

### DNA transfection and recovery of mutant viruses

The full-length cDNA PRRSV clones, pAPRRS and derivatives, and nonreplicative control pAS were purified by the QIAprep Spin Miniprep Kit (Qiagen) and quantified by spectrophotometry and 1% agarose gel electrophoresis as described previously [30,31]. 70% confluent BHK-21 cells seeded in six-well plate were used for transfection, Lipofectamine™ LTX and Plus Reagent (Invitrogen) were used, according to the manufacturer's specifications, with minor modification. The supernatants were collected at 24 hpt, aliquoted and designated as passage 0 (P0) of the rescued viruses, and stored at -80°C for further analysis. The infectivity of the mutants was tested by infecting fresh MARC-145 cells, and five passages (P1-P5) were conducted as previously described [32].

### Indirect immunofluorescence assay (IFA)

The transfected BHK-21 cell monolayer was used for assessing viral protein expression as previously described [33]. Briefly, at 24 hpt, the cell monolayer was fixed with cold methanol for 10 minutes and then blocked with 0.1% BSA for 30 minutes, followed by incubation at 37°C for 2 hours with monoclonal antibody against N protein of type II PRRSV (kindly provided by Dr. Ying Fang at South Dakota State University). The cells were incubated at 37°C for 1 hour with Alexa Fluor 568-labeled goat anti-mouse IgG (H+L) (1:800 diluted, Invitrogen). The stained cell monolayer was visualized under an Olympus inverted fluorescence microscope.

### Northern blot analysis

Northern blot was performed according to the NorthernMax kit (Ambion, Austin, TX). 2 μg mutant plasmids were transfected into MARC-145 cells and intracellular RNAs were isolated from transfected cells at 48 hpt using TRIzol^® ^Reagent (Invitrogen). The RNAs were separated on 1% denatured agarose gels using Agarose-LE (Ambion), blotted onto a nitrocellulose membrane by an N-specific probe PR3. The hybridization was conducted at 42°C overnight, followed by washing with low/high-stringency buffers, wash buffers and blocking buffers. The membrane was incubated with the chemiluminescent substrate CDP-STAR and exposed the blot image on film overnight.

### Viral plaque assay

MARC-145 cells in six-well plates were infected with WT and rescued viruses (P1) at 0.01 multiplicity of infection (MOI). After 1 hour adsorption at 37°C, the cell monolayer was washed and replaced with 5 ml of equal volume of mixture of MEM containing 2% FBS and 1% low melting agarose (Cambrex). After the gel overlay solidified, the plate was reversely (top side down) placed into an incubator at 37°C with 5% CO_2_. At 4 days post infection (dpi), the plaque was visualized by crystal violet staining. The plaque size was determined with a millimeter ruler.

### Multi-step growth curve

To assess the viral growth kinetics, MARC-145 cells in six-well plates were infected with the rescued viruses (P1) at 0.01 MOI as described previously [32]. Briefly, 200 μl of supernatant was harvested at 6, 12, 24, 36, 48, 60, 72, 84, 96, 108, and 120 hpi. Virus titration was performed by viral plaque assay. Each experiment was independently repeated three times and SD was calculated.

### Reverse transcription PCR (RT-PCR) and nucleotide sequencing

Viral RNAs from transfected or infected cell supernatants were extracted using a QIAamp Viral RNA Mini Kit (Qiagen) according to manufacturer's instruction. 1 μg of viral RNA was used for reverse transcription with 10 μM primer Qst, an anchored-poly(T) primer (Table 1), using a RT kit (Takara). Oligonucleotide F-6 and R-683 (complementary to nt 661-683) were forward and reverse primers. The RT-PCR products were gel-purified by QIAgen PCR purification kit (Qiagen), and submitted to direct sequencing by a commercial supplier (Shanghai Sangon Inc.). When necessary, the RT-PCR product was cloned in pGEM-T vector (Promega), followed by nucleotide sequence determination. The sequencing primer information is available from the authors upon request. Nucleotide sequence analysis was conducted with the DNASTAR program (Lasergene Package).

### Detection of (-) gRNAs and sg mRNAs by RT-PCR

BHK-21 cells in six-well plates were transfected by the mutant plasmids as described above. Total cellular RNAs were isolated from the tranfected cells at 24 hpt using TRIzol^® ^Reagent (Invitrogen). RNAs were suspended in DNase/RNase-free water, and quantified by NanoDrop^® ^ND-1000 (Thermo Fisher Scientific Inc.). To eliminate the transfected input DNA, the RNA preparation was further treated with 2 U RNase-free recombinant DNase I for 30 minutes at 37°C by using the DNA-free Kit (Ambion), followed by re-suspension in RNase-free H_2_O. RT-PCR was employed to detect the (-) gRNAs and sg mRNAs using specific primers. As an internal control, the housekeeping β-actin mRNA was also performed on the same RNA preparations using primer pairs F-actin and R-actin (Table 1).

For (-) gRNA detection, forward primer F-6 (nt 6-26, GQ330474, Table 1) was used for first-strand cDNA synthesis with reverse transcriptase Superscriptase III (Invitrogen) from 2 μg of total RNAs. The resultant cDNA was treated with 2 μg RNase A (Invitrogen) for 30 minutes at 37°C to remove the remaining RNAs, followed by inactivation of RNase A by heating at 95°C for 10 minutes. 2 μl of cDNA was used for primary PCR with primer pair F-6 and R-683 (complementary to nt 661-683) for 30 cycles of denaturation at 95°C for 30 seconds, annealing at 58°C for 30 seconds, and extension at 72°C for 30 seconds. Nested PCR with internal primer pair F-12 (nt 12-32) and R-343 (complementary to nt 323-343) were performed using 2 μl of 1000-fold diluted primary PCR products, with the same PCR cycle parameters.

For (+) sg mRNA7 detection [31], the cDNA was synthesized by RT primer Qst (Figure 2A right panel, Table 1). The (+) sg mRNA7 was subsequently amplified to obtain the leader-body junction containing fragment using oligonucleotide F-12 (nt 12-32) and R-15284 (complementary to nt 15262-15284). Thirty cycles of PCR were performed as follows: 95°C denaturation for 30 seconds, annealing at 63°C for 30 seconds, and extension at 72°C for 30 seconds. (-) sg mRNA7 was amplified in the similar manner, except that the RT primer was F-12 (Figure 2A right panel, Table 1). Each experiment was independently repeated three times.

### RNA secondary structure analysis

RNA secondary structure were predicted using the MFold web server version 2.3 [29], while the VIENNA RNAFOLD program and RNASTRUCTURE 5.0 were also used to compare the predictions. All predictions were conducted under default conditions of the software. The predicted secondary structure was modified by RNAviz 2.0 (http://rnaviz.sourceforge.net/).

## Results

### PRRSV 5'UTR displays inter-genotypically conserved high order structure, despite with distantly related primary sequences

The primary sequences between type I and type II PRRSV 5' UTRs show about 50% sequence identity [4,27]. We analyzed the possible high order structure of the PRRSV 5'UTR by the MFold program [29]. By multiple nucleotide sequence alignment of the 5' genomic ends of nine representative type II and five type I PRRSV strains (detailed in the Material and Methods section), consensus primary sequences were generated for the 5'-proximal 246 nt and 280 nt of type II PRRSV of type I PRRSV, respectively. The high order structures of the two types of PRRSV 5'-proximal region were strikingly similar and characterized by six major putative helical stem-loops, which we arbitrarily designated as E-SL1-5 for type I PRRSV and N-SL1-5 for type II PRRSV, respectively (Figure 1A i and 1A ii). It should be noted that E-SL1 in type I PRRSV could be separated into two minor stem-loops, named E-SL1a and E-SL1b, which corresponded to N-SL1 in type II PRRSV. Similarly, N-SL4 in type II PRRSV also could be divided into two minor stem-loops, named N-SL4a and N-SL4b, which corresponded to E-SL4 in type I PRRSV. The prominent N/E-SL5 resembles that of the EAV LTH structure demonstrated by Van den Born et al. [4,27]. In addition, we conducted similar MFold analysis to the consensus sequences generated by comparison of 237 available GenBank deposited type II PRRSV sequences corresponding to the first 246 nucleotides of the APRRSV genome, not unexpectedly, this set of structures held true except that minor changes in the flanking linear strand region of SL4a (data not shown).

Among the five stem-loops, SL2 is a highly conserved between different genotypes of PRRSV, despite the primary sequences of N-SL2 and E-SL2 showing only 48% similarity (Figure 1A i, 1A ii). A "ternary-turn loop" is located at the top of the SL2, and the stem consists of G-C rich base pairs (9/11 in E-SL2 and 8/10 in N-SL2). Compared with the type II consensus sequences of N-SL2, APRRSV strain (GenBank: GQ330474), the backbone used in this study, contains a co-variation C_56_-G_62 _that was replaced with base pairs U_56_-A_62_. In addition, there were two single-nucleotide bulges in both SL2s. Both the VIENNA RNAFOLD program and RNASTRUCTURE 5.0 software predicted the same robust secondary structures in the 5'-proximal region of the two types of PRRSV.

### The loop size of N-SL2 is irrelevant to PRRSV replication

To investigate the function of the N-SL2 sequence and structure, site-directed mutagenesis was performed on a DNA-launched infectious cDNA clone pAPRRS (Figure 2A), a type II PRRSV that was under the control of the CMV promoter [28]. In the first set of mutants, the size of the "ternary-turn loop" was increased to 8 nt by disruption of the upper part of the stem in N-SL2. In mutant L-LL, G_61_-A_62_, 2 nt at the 3' side of the loop, were changed to C_61_-U_62 _and became identical to the nucleotides on the opposite side (Figure 2B, upper panel). The same approach was used for the 5' side of the N-SL2 stem in mutant L-RR (U_56_-C_57 _to A_56_-G_57_). It should be pointed out that the base-pairs in the upper part of the stem and the A_52 _budge was also disrupted for both L-LL and L-RR. The normal size of the loop was restored in mutant L-RL by combining these two mutations in both sides of the loop. The CMV promoter-driven mutant plasmids were transfected into BHK-21 cells as described in the Materials and Methods section. Total RNAs of the plasmids transfected cells were isolated by TRIzol^® ^Reagent at 24 hpt.

To analyze the viral mRNA profiles in the transfected cells, we first established the RT-PCR methods to eliminate the possible interferences from the input DNA and the RNA transcript generated by the CMV promoter. Total RNAs of cells transfected by the nonreplicative pAS containing large internal deletion (nt 1688-13118, GQ33047) of pAPRRSV, which also served as WT control, were utilized for verifying the treatments by DNase I, RNase A, or the combination of both. The resultant RNA samples were then amplified by nested PCR for the presence of the (-) gRNA intermediate. Treatment by either DNase I or RNase A was not enough to eliminate the input DNA and CMV-promoter driven RNAs, as the expected PCR product (321bp) from pAS and WT all could be seen (Figure 2C lane 4, 5 and 8, 9). On the other hand, treatment by DNase I alone but not RT and RNase A was demonstrated that DNase I treatment was successful (Figure 2C lane 6, 7). After serial treatment prior to RT by 2U DNase I and post RT reaction by 2 μg RNase A, no PCR product of the expected size was amplified from the transfected cells of nonreplicative control pAS, but the expected PCR product for WT was still amplified (Figure 2C lane 10, 11). These results showed that the transfected DNAs and the CMV-driven RNAs were completely eliminated, and thus validate that RT-PCR method for detecting the presence of newly synthesized viral RNAs, if any. Utilized this protocol, we investigated the presence of the (-) gRNA. Agarose electrophoresis results revealed that the primary PCR with F-6 and R-683 was barely seen (data not shown), however, the nested PCR with internal primer pair F-12 and R-343 revealed the presence of (-) gRNA from the transfected cells by L-LL, L-RR, and L-RL (Figure 2D), with abundance comparable to that of the APRRSV. These results suggested that the loop size can be variable from 3 nt to 8 nt without deleterious effect on (-) gRNA synthesis.

We proceeded to detect the presence of sg mRNA7 and its (-) sg mRNA7 template by subgenomic mRNA specific RT-PCR from the total RNAs of the transfected cells. As illustrated in Figure 2D, the presence of both (+) sg mRNA7 and (-) sg mRNA7 in transfected cells by the loop mutants L-LL/RR/RL were detected at a comparable level to WT, indicating that the disrupted SL2 loop, and the absence of the upper part of the stem and the A_52 _budge, did not impair sg mRNA synthesis.

**Figure 3:**
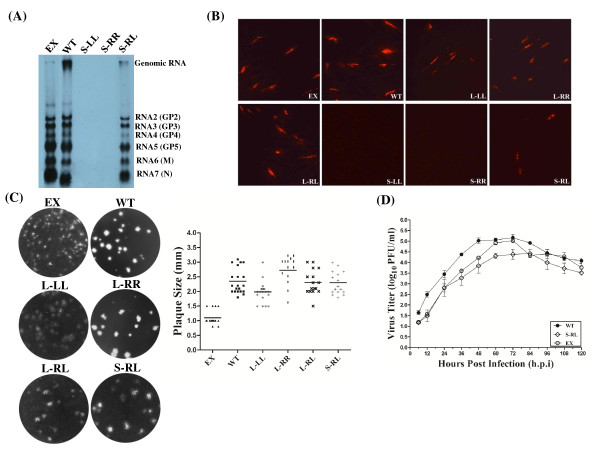
**Structural protein expression and phenotypic properties of the mutant viruses**. (A) Northern blot analysis of mutant RNAs isolated at 48 hours post transfection from MARC-145 cells transfected with WT, EX, S-LL, S-RR and S-RL plasmids. (B) PRRSV N protein expression of WT and mutants in transfected cells. Expression of N protein was visualized by immunofluorescence staining with anti-N antibody at 24 hours post-transfection. (C) Viral plaque morphology assay. 0.01 MOI of P1 supernatants were inoculated in fresh MARC-145 cells and covered by MEM containing 2% FBS and 1% low melting agarose, and the plaques were visualized at 5 days post infection by crystal violet staining. The plaque sizes of the WT (■) and mutant EX (▲), L-LL(*), L-RR(▏), L-RL(╳), and S-RL (**+**), were measured by a millimeter ruler after monolayers were stained with crystal violet. The bars represent the average plaque diameters. (D) Viral multi-step growth curves. MARC-145 cells infected at an MOI of 0.01 with the P1 passage parental virus and mutant viruses and harvested at the indicated time points. The virus titers were determined by plaque assay and the results were mean values from three independent experiments. Viral titers were expressed as log_10 _PFU/ml.

Since SL2 is located upstream of the sg mRNA7 initiation codon, we then analyzed its possible effect on structural protein expression. The expression of N protein was measured by indirect immunofluorescence assay (IFA) of transfected BHK-21 cells with antibodies specific for nucleocapsid protein (N). As shown in Figure 3B, IFA revealed that the loop size mutants L-LL/RR/RL could detect the N protein in cells transfected with plasmids containing these mutations. Taken together, these results demonstrated that the loop of N-SL2 was not crucial for viral genomic RNA replication, sg mRNAs synthesis and translation of structural protein.

To investigate further the possible effect of SL2 mutation on viral properties, the transfected BHK-21 supernatants (P0) were inoculated into fresh MARC-145 cells, from which viral plaque assay and multi-step growth kinetics of the rescued mutant viruses were determined. The average diameter of the WT viral plaque was 2.4 mm, while the recovered mutant viruses displayed virtually the same size including L-LL (2.0), L-RR (2.6), L-RL (2.4) (Figure 3C). We further assessed the multi-step growth kinetics of the recovered mutant viruses in P1 and compared them with those of the parental WT virus (Figure 3D). The WT virus achieved the peak titer of 5.16 log_10 _PFU/ml at 72 hpi, while the mutant viruses L-LL, L-RR, and L-RL displayed similar virus growth kinetics and peak titer (data not shown). These results demonstrated that the loop size, ranging from 3 to 8 nt, and the two base-pairs and the budge in the upper part of the stem of the SL2 could be irrelevant to the PRRSV virus replication cycle.

### The stem structure of N-SL2 is crucial for PRRSV sg mRNA synthesis

We further investigated the possible roles of N-SL2 stem. Using the same approach as for loop mutants, we created mutant S-LL by replaced the 3' side of the whole stem of N-SL2 with the 5' side of the stem, including two single-nucleotide bulges to disrupt the whole structure of N-SL2 (Figure 2B, lower panel). The S-RR mutant was also constructed by substitution of the 3' for the 5' side of the stem. By combination of the two mutations in the double mutant S-RL, the stem of N-SL2 was restored by the same base pairs. Upon transfection into BHK-21 cells and subsequent passage in MARC-145 cells, S-LL and S-RR showed no evidence of infectivity, which was readily detected in the S-RL mutant. These results demonstrated that the N-SL2 stem was crucial for virus viability.

We next analyzed the viral RNA profiles using the same RT-PCR as described for the loop mutants. Again, the (-) gRNA synthesis was not significantly affected by the structural alteration in S-LL, S-RR and S-RL, comparable level of (-) gRNA with WT were detected (Figure 2D). However, both (-) and (+) sg mRNA7 synthesis were abolished in S-LL and S-RR (Figure 2D). Surprisingly, the double mutant (S-RL) did restored the synthesis of (+) sg mRNA7, albeit at a relatively low level that the WT, implying that the sg mRNA synthesis was affected to some extent. Moreover, the (-) sg mRNA7 in S-RL was undetectable using the described RT-PCR procedure. To analyze the viral RNA patterns, the plasmids S-LL, S-RR and S-RL were transfected into MARC-145 cells, and intracellular RNAs harvested at 48 hpt were subjected to northern blot analysis with the PR3 probe. The result confirmed that disruption of stem structure in S-LL and S-RR resulted in loss of any viral RNAs including the (-) gRNA detected by the nested RT-PCR procedure. The failure to detect the genomic RNAs might be attributed to the low sensitivity of northern blot procedure, the low efficiency of genomic RNA transfer to the membrane by traditional capillary method, or the combination of both. Nonetheless, S-RL indeed restored genomic RNA synthesis as well as all of sg mRNAs (Figure 3A).

IFA also revealed that N protein could not be detected at all in cells transfected with S-LL and S-RR mutants, possibly due to the lack of sg mRNA. However, the N protein expression was restored in the S-RL transfected cells (Figure 3B). These results clearly indicated that the stem structure of N-SL2, but not the "ternary-turn loop", is the crucial functional structure for viral sg mRNA synthesis. However, the stem restored mutant S-RL showed a lower viral titer at every time point of infection, with the peak titer of 4.42 log_10 _PFU/ml at 84 hpi, which was delayed by 12 hours compared with WT (Figure 3D). These results suggested that the N-SL2 stem mutation rendered the virus replication in the cultured cells less fit, but nonetheless was crucial for virus viability.

### Type II 5'UTR SL2 could be structurally and functionally replaced by that of type I

Despite the primary sequences of N-SL2 and E-SL2 sharing only 48% similarity, SL2 is an inter-genotypically conserved structure in both PRRSV types (Figure 1A). Specifically, E-SL2 was predicted to have one more base pair than N-SL2, and 11 nucleotides out of the 25 comparable nucleotides were different (Figure 1B). This led us to investigate whether N-SL2 could be replaced by E-SL2. *In silico *analysis revealed that N-SL2 could be structurally replaced by E-SL2 without altering the overall structure of type II PRRSV 5' end (Figure 1A iii). Utilizing site-directed PCR mutagenesis, plasmid EX that contained E-SL2 in the type II PRRSV backbone was generated.

To investigate genome replication and sg mRNA transcription in more details, intracellular RNAs were isolated from transfected BHK-21 cells and analyzed by PCR analysis. The presence of the (-) gRNA, (+) sg mRNA7 and (-) sg mRNA7 in EX was detected at comparable levels with WT (Figure 2D), suggesting that the genome replication and sg mRNAs synthesis were not markedly affected by the substitution of SL2. The transfected supernatant was inoculated into fresh MARC-145 cells, and the total RNAs were extracted for northern blot analysis. As shown in Figure 3A, EX actually produced more sg mRNAs but less gRNA than those of WT. At this moment, we are not sure whether the observed abundance discrepancy between the sg mRNAs and gRNAs was caused by imbalanced membrane transfer, or the E-SL2 substitution indeed shifted the ratios of gRNA versus sg mRNAs. Nonetheless, the substitution of E-SL2 did restore viral RNA synthesis that was absent in the disrupted SL-2 stem mutants S-LL and S-RL, indicating that E-SL2 could structurally and functionally replace the N-SL2 counterpart in the heterologous genomic background. At the same time, the N protein expression was also not impaired in the transfected BHK-21 cells (Figure 3B), measured by IFA with anti-nucleocapsid antibody.

The plaque phenotypes of WT and chimeric virus EX in P1 were distinctly different (Figure 3C). Average plaque diameters of EX were only 1.1 mm, which were only 40% of those in the corresponding WT virus. These results suggested that the phenotypic properties of EX were affected by the substitution of SL2 between the two genotypes. The titer of EX was consistently 10-fold lower before reaching a peak of 5.03 log_10 _PFU/ml at 72 hpi (Figure 3D), compared to 5.16 log_10 _PFU/ml at 72 hpi for WT.

### Stem integrality of N-SL2 was essential for sg mRNA transcription

**Figure 4:**
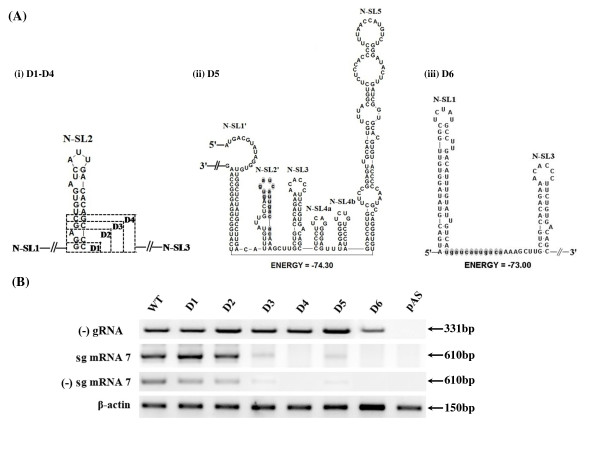
**Mutagenesis of the serial deletion of base pairs in the N-SL2 stem**. (A) RNA secondary structure prediction of the mutants. (i) Schematic representation of the predicted secondary structure of the mutants, D1-D4. The dashed boxes indicate the increasing base pair deletions from the bottom of N-SL2. (ii) RNA secondary structure prediction of the mutant D5. The remaining nucleotides of N-SL2 are highlighted in gray. N-SL1' and N-SL2' represent the regenerated stem-loop structures after the deletions. (iii) Predicted secondary structure of mutant D6. The remaining nucleotides of N-SL2 are indicated by gray shading, which disappeared and became linear nucleotides. (B) RT-PCR analysis of the mutants D1-D6 as described in Figure 2.

To gain further insight into the structure and function of N-SL2, we conducted serial base-pair deletions from the bottom to the top of the stem, and the shortened-stem mutants were designated as D1 to D6, respectively. The schematic structure is shown in Figure 4A. Mutant D2 included two base pairs and one single-nucleotide bulge deletion. Secondary structure folding by MFold [29] indicated that mutants D1-D4 contained an overall N-SL2 structure, except that the stem was gradually shortened by the increasing number of base-pair deletions (Figure 4A i). However, mutant D5 created a predicted new stem-loop 2 (N-SL2', Figure 4A ii) by reconfiguration of the upstream sequences of N-SL1. Moreover, N-SL1 was replaced by a similar structure designated as N-SL1', in which the 5'-end sequences of N-SL1 interacted with 3'-end sequences of N-SL5. For mutant D6, N-SL2 completely disappeared, and it formed linear nucleotides (Figure 4A iii).

The mutant plasmids D1-D6 were transfected into BHK-21 cells and further RT-PCRs were used to detect the (-) gRNAs, (+) sg mRNA7 and (-) sg mRNA7 of mutant viruses. It was shown that mutants D1-D6 produced (-) gRNA as the WT virus (Figure 4B). Furthermore, mutant D1 and D2 produced similar levels of (+) sg mRNA7 and (-) sg mRNA7, whereas D3 only produced lower level of these sg mRNAs. However, in mutant D4 transfected cells, neither sense nor antisense sg mRNA7 was detectable, which suggested that the deletions severely affected sg mRNA transcription. To our surprise, mutant D5 that contained one more base pair deletion than D4 restored synthesis of (+) sg mRNA7 and (-) sg mRNA7, albeit at a relatively low level. In addition, when N-SL2 completely disappeared in mutant D6, sg mRNA synthesis was also totally inhibited, as in D4. Further *in silico *analysis revealed that D5 regenerated a predicted N-SL-like stem loop, designated as N-SL2', by the remaining N-SL2 sequence formed base-pairing with the upstream N-SL1 sequence (Figure 4A ii). On the other hand, the N-SL-1 structure was restored by the 5'-proximal sequences tertiary interact with the downstream sequence in the ORF1 region. These results demonstrated that stem integrality of the N-SL2 structure was essential for the synthesis of sg mRNA.

**Figure 5:**
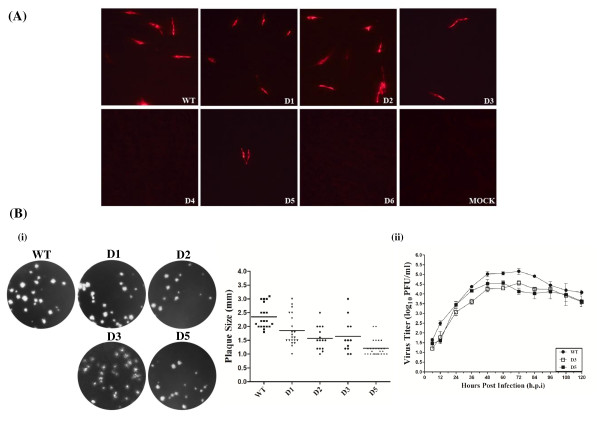
**Structural protein expression and phenotypic properties of the stem deletion mutant viruses**. (A) Intracellular N protein expression of WT and mutant viruses. BHK-21 cells were transfected with plasmids of WT and mutants D1-D6 as indicated in Figure 3. Expression of N protein was visualized by immunofluorescence staining at 24 hours post-transfection. (B) Viral plaque morphology and growth curves of the mutant viruses D1-D6 as described in Figure 3.

IFA revealed that the expression level of N protein was correlated to the presence or absence of sg mRNA7 (Figure 5A). In mutants D4 and D6, positive cells were completely absent, probably because that the sg mRNA7 synthesis of these mutants was completely inhibited by the deletions. However, mutant D5 restored the expression of N protein, albeit the positive numbers of cells were significantly lower than for the WT, D1, D2 and D3. Taken together, these results revealed that the stem integrality of N-SL2 played a key role in the process of the discontinuous sg mRNA transcription.

To investigate the possible effects of N-SL2 mutation on viral properties, plaque size and growth kinetics of the recovered mutant viruses in P1 were determined (Figure 5B). Average plaque diameters of D1, D2, D3 and D5 viruses were 1.8, 1.5, 1.6 and 1.3 mm, respectively (Figure 5B i), which were smaller than the WT plaque diameter at 2.4 mm. This indicated that the structural changes in the stem of N-SL2 adversely affected the phenotypes (at least the plaque size) of the mutant viruses. In the growth kinetics analysis, D1 and D2 achieved maximal titers of 5.06 and 5.02 log_10 _PFU/ml at 72 hpi (data not shown), respectively, which were at similar levels to the WT. However, the mutant D3 titer was 10-fold lower than that of the WT, and only reached a peak of 4.57 log_10 _PFU/ml at 72 hpi, compared with 5.16 log_10 _PFU/ml at 72 hpi for the WT (Figure 5B ii). Intriguingly, the mutant D5 reached peak titer of 4.57 log_10 _PFU/ml at 60 hpi, which was 12 hours earlier that D3, yet quickly fell below the level of the latter afterwards. These results suggested that the deletion at the N-SL2 stem bottom significantly decreased the growth level, probably because that the sg mRNA synthesis was affected by the manipulation of SL2.

### Genetic stability of the rescued mutant viruses

**Figure 6:**
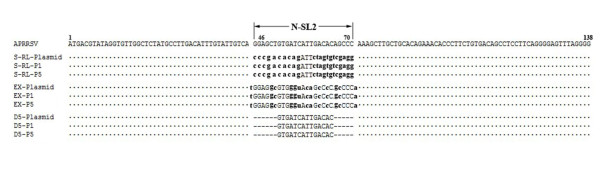
**Genetic stability of the rescued mutant viruses**. DNASTAR v7.1 program (Lasergene Package) was used to conduct nucleotide sequence alignment of the rescued mutant viruses. Dots indicated that the residues match APRRSV (GQ330474) exactly. All mutant sequences were shown as lowercase. Short lines meant the deletions in D5 mutant viruses comparing with APRRSV. The numbers indicated the sequence positions of APRRSV.

To assess the genetic stability of the mutant viruses during subsequent passages in MARC-145 cells, we determined genomic sequences of the full-length of all mutant viruses in P1 and P5 as described previously (Sun et al., 2010). RT-PCR products were subjected to direct nucleotides sequencing, and such population genomic sequences revealed no significant changes except the engineered mutations in S-RL, EX and D5. As shown in Figure 6, all of the original site-directed mutations in S-RL, EX and D5 plasmids were retained in the recovered viruses (P1 and P5), which indicated that the mutant viruses were genetically stable.

## Discussion

It has been established that various *cis*-acting RNA sequences and structural elements that reside in the 5' UTR of the genome of positive-strand RNA viruses are vital for controlling viral replication processes, including genome replication, sg mRNA transcription, genome encapsulation, and/or translation. Here, we present genetic evidence to support that the predicted N-SL2 stem-loop structure PRRSV type II 5' UTR is essential for viral replication, and particularly sg mRNA synthesis. This inter-typically conserved stem-loop structure can be replaced by heterologous E-SL2 of the type I PRRSV 5' UTR, although only limited primary sequence identity exists. We further demonstrated that the N-SL2 functional domain resided in its stem structure rather than the ternary-loop. These results may lead to further investigation and better understanding of the life cycle of PRRSV, which is one of the biggest threats to the swine industry.

**Figure 7:**
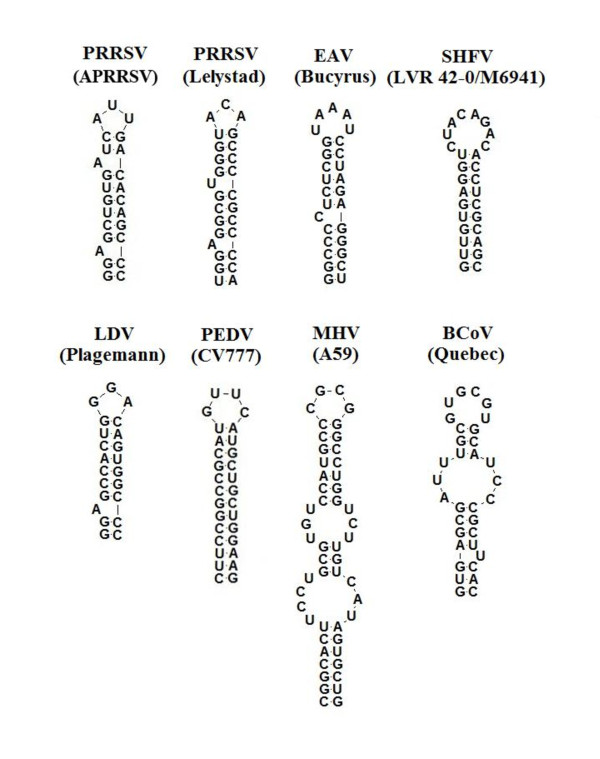
**Similar N-SL2 RNA secondary structure can be predicted from arteriviruses and coronaviruses**. Arteriviruses presented are PRRSV strains APRRSV (GenBank: GQ330474) and Lelystad (M96262), SHFV strain LVR 42-0/M6941 (NC_003092), and LDV strain Plagemann (NC_001639). For coronaviruses, presented strains were PEDV strain CV777 (NC_003436), MHV strain A59 (NC_001846) and BCoV strain Quebec (AF220295).

We showed that the SL2 was structurally and functionally conserved in two genotypes of PRRSV. Next, we investigated if a similar structure could also be found in other arteriviruses and coronaviruses? Snijder and co-workers have done extensive studies on EAV, the prototypic species of *Areriviridae *[4,27]. By combining *in silico *analysis and structure probing, they have proposed that the structure model for the 5' proximal EAV genome consists of a series of stem-loop structures that range from A to J. The third stem-loop structure (C) also contains a G-C rich base pair stem (6/8) [4]. Further secondary structure MFold predictions of the 5' UTR sequences of other arteriviruses including lactate dehydrogenase-elevating virus (LDV, SL2) and simian hemorrhagic fever virus (SHFV, SL3) were conducted. Surprisingly, a similar structure (SL2) that comprises a ternary-loop and a G-C rich base pair stem (7/9) exists in LDV (Figure 7), which suggests that SL2 is a structurally conserved element in arteriviruses. Whether these similar structures also regulate viral sg mRNA synthesis is worthy of further investigation. We then made a similar comparison with 5' UTR structure models of various coronaviruses, another family member of the *Nidovirales *[14,15,34,35]. Although significantly different structural models exist among the coronaviruses, similar G-C rich base pair stem-loops can also be predicted in porcine epidemic diarrhea virus (PEDV) (7/13, SL4), MHV (10/17, SL4) and bovine coronavirus (BCoV) (7/10, SL1), which suggests that N-SL2 is a structural element that is conserved in the order *Nidovirales *(Figure 7).

We demonstrated here that the structurally conserved SL2 was vital for PRRSV sg mRNA transcription. It has been structurally and functionally confirmed that the LTH is an independent transcription-regulating element in the EAV system [4,27], and such an LTH is indeed shared by other arteriviruses. Whether the LTH and SL2 described in our study are independent or cooperative *cis*-acting elements that control sg mRNA transcription is still unknown, and further functional study of PRRSV LTH is under way.

We observed a very interesting phenomenon that mutant D5 can restore viral infectivity through creating an alternative N-SL2-like structure, N-SL2', which further signifies the high-order structural nature of N-SL2. The alternative N-SL2' was generated by rearranging the remaining SL2 sequences after D5 deletions and part of the upstream N-SL1 sequences. Meanwhile, the 5' proximal sequences of N-SL1 interacted with 3'-end sequences of N-SL5 to form a tertiary alternative N-SL1', which suggests that tertiary structural regulating elements do play a role in PRRSV RNA synthesis control. It is of interest to study whether N-SL1 and the alternative N-SL1' regulate sg mRNA transcription, and if this is that case, how it would work in synchrony with SL2 and/or LTH. The generation of N-SL1' seems to be based on base pairing between the partially complementary sequences. However, we cannot rule out the possibility that such tertiary interactions could be facilitated by RNA-protein-RNA interaction, which has been established as a major way for viral component conformational changes. For instance, the 5' UTR of severe acute respiratory syndrome virus genomic RNA can be specifically bound by the 3a protein, and a 55-kDa cellular protein has been found to bind to MHV 5' genome terminus [36,37]. Precedents in other positive-strand RNA viruses [38-41] lead us to speculate that stem-loop 2 or other stem-loop structures in PRRSV may bind viral or cellular proteins in the positive-strand. In mutant D6, N-SL2 was completely destroyed and changed to linear nucleotides. We speculate why mutant D6 could not recreate an alternative stem-loop 2 in the same way as mutant D5. It is possible that the free energy of the secondary structure will make the difference. In fact, the single-strand form of N-SL2 would make the whole 5' terminal region more stable (free energy = -73.00 kcal/mol), relative to creating a new stem-loop 2 (free energy = -72.60 kcal/mol). In addition, the lengthy deletion of D6 mutant may bring about overt changes in the overall structure of the 5' proximal region and/or the whole genome, so that any restoration is impossible.

Upon entry into the cells, arterivirus replication cycle starts with uncoating the translational of the nonstructural proteins that ultimately assemble into replication and transcription complex (RTC). RTC is responsible for initiation of negative-strand genomic RNA and subgenomic mRNA templates, based on which progeny genomic RNAs and mRNAs for structural proteins were synthesized. The translated structural proteins are then assembled into virions, starting with encapsidation of progeny genome by encapsidation (Snijder and Mulenberge, 1998). Nidovirus is believed to adopt ribosomal leaky-scanning model for the expression of the mRNAs [5] The Snijder group has provided experimental evidences for such leaky scanning translation for EAV mRNAs, for which the 5' UTR probably has no function [42]. In this study, we tested each mutant for non-structural protein 2 (nsp2) translation levels, and found no significant difference among the different mutants (data not shown). Nevertheless, it is intriguing that none of the SL2 mutations described here affected anti-genomic RNA template synthesis, yet the subgenomic RNA synthesis in both senses were affected. Locating on the 5' -proximal genomic ends, SL2 may exert regulatory function via interaction with the 3' -end via RNA-RNA, or RNA-protein-protein-RNA interactions. Such genomic end long-range interactions have been well documented in flaviviruses [43-47]. It remains unknown if such genomic cyclization could occur in arteriviruses, but it was reported that both PRRSV and SHFV 3' UTR interact with host cell proteins.

The genome packaging signals (Ps) of arteriviruses are unknown. Because that PRRSV could package defective (heteroclite) RNAs, consisting of PRRSV genomic termini, Yuan et al. proposed that the Ps for PRRSV genome possibly localizes in the 5' proximal ends [48]. We presented here that SL2 mutations could produce similar intracellular viral RNAs, yet the phenotypic properties varied. It is reasonable to speculate that the SL2 mutation could brought about change of the local or overall genomic high-order structure, such that the genomic encapsulation or genomic versus mRNA ratio were altered, as described in EX.

## Conclusion

Here, we have focused on the PRRSV 5' UTR to gain further insight into the structure and function of it. By *in silico *analysis of two types of PRRSV 5' UTR, we presented a conserved stem-loop structure between them. Firstly, this structure could be exchanged between different genotypes of PRRSV. Secondly, through a series of deletions and substitutions in the full-length infectious clone of PRRSV type II, we determined the functional significances of the potential stem-loop (SL) structure in PRRSV 5' UTR. Finally, we presented evidence that this SL structure was a key structural element for PRRSV sg mRNAs synthesis.

## Competing interests

The authors declare that they have no competing interests.

## Authors' contributions

JL designed and performed the study. JL, FG and PL drafted the manuscript. YL and TL revised the manuscript. ZW and CL participated in statistical analysis. HZ helped to carried out the RT-PCR and helped to analyze the results. SY conceived the study and critically reviewed the manuscript. All authors read and approved the final manuscript.
